# Functional proteomics outlines the complexity of breast cancer molecular subtypes

**DOI:** 10.1038/s41598-017-10493-w

**Published:** 2017-08-30

**Authors:** Angelo Gámez-Pozo, Lucía Trilla-Fuertes, Julia Berges-Soria, Nathalie Selevsek, Rocío López-Vacas, Mariana Díaz-Almirón, Paolo Nanni, Jorge M. Arevalillo, Hilario Navarro, Jonas Grossmann, Francisco Gayá Moreno, Rubén Gómez Rioja, Guillermo Prado-Vázquez, Andrea Zapater-Moros, Paloma Main, Jaime Feliú, Purificación Martínez del Prado, Pilar Zamora, Eva Ciruelos, Enrique Espinosa, Juan Ángel Fresno Vara

**Affiliations:** 1 Molecular Oncology & Pathology Lab, Institute of Medical and Molecular Genetics-INGEMM, La Paz University Hospital-IdiPAZ, Madrid, Spain; 2 Biomedica Molecular Medicine SL, Madrid, Spain; 3 Functional Genomics Center Zürich, University of Zürich/ETH Zürich, Zürich, Switzerland; 4 Department of Statistics, Biostatistics Unit, La Paz University Hospital - IdiPAZ, Madrid, Spain; 5 Operational Research and Numerical Analysis, National Distance Education University (UNED), Madrid, Spain; 6 Medical Laboratory Service, La Paz University Hospital Health Research Institute-IdiPAZ, Madrid, Spain; 7 Department of Statistics and Operations Research, Faculty of Mathematics, Complutense University of Madrid, Madrid, Spain; 8 Medical Oncology Service, La Paz University Hospital-IdiPAZ, Madrid, Spain; 9 Medical Oncology Service, Basurto Hospital, Bilbao, Spain; 10 Medical Oncology Service, Hospital 12 de Octubre (i+12) Health Research Institute, Madrid, Spain

## Abstract

Breast cancer is a heterogeneous disease comprising a variety of entities with various genetic backgrounds. Estrogen receptor-positive, human epidermal growth factor receptor 2-negative tumors typically have a favorable outcome; however, some patients eventually relapse, which suggests some heterogeneity within this category. In the present study, we used proteomics and miRNA profiling techniques to characterize a set of 102 either estrogen receptor-positive (ER+)/progesterone receptor-positive (PR+) or triple-negative formalin-fixed, paraffin-embedded breast tumors. Protein expression-based probabilistic graphical models and flux balance analyses revealed that some ER+/PR+ samples had a protein expression profile similar to that of triple-negative samples and had a clinical outcome similar to those with triple-negative disease. This probabilistic graphical model-based classification had prognostic value in patients with luminal A breast cancer. This prognostic information was independent of that provided by standard genomic tests for breast cancer, such as MammaPrint, OncoType Dx and the 8-gene Score.

## Introduction

Breast cancer is a major health issue in developed countries. Early diagnosis and the use of adjuvant therapies have contributed to improve survival; nevertheless, 87,000 women died of breast cancer in the European Union in 2011^1^. Knowledge of the molecular biology of breast cancer has recently challenged the way in which oncologists make decisions about systemic treatment^2^.

Breast cancer is a heterogeneous disease comprising a range of entities with various genetic backgrounds. Clinical decisions are currently based on classical factors, such as the extent of the disease and the expression of hormonal receptors and human epidermal growth factor receptor 2 (HER2). Genomic classifications have also been described, the better-known encompassing four major categories: luminal A, luminal B, basal-cell and HER2-enriched^3^. Most patients included in the categories of estrogen receptor-positive (ER+)/HER2-negative (HER2-) disease with luminal A breast cancer have a favorable prognosis; however, some eventually relapse, which suggests some heterogeneity within these categories. Patients in the categories of triple-negative disease — i.e., no expression of hormonal receptors, HER2- or basal-cell disease — have a poorer prognosis^4, 5^.

In recent years, proteomic approaches have been incorporated into the study of clinical samples as a way to complement the information provided by classical factors and genomics. Mass spectrometry-based proteomics has emerged as preferred component of a strategy for discovering diagnostic and prognostic protein biomarkers as well as for establishing new therapeutic targets^6^. Although these investigations are encouraging^7, 8^, the number of tumor biomarkers discovered with this approach is still limited^9^. MicroRNAs are key regulators in the genesis and progression of cancer. MicroRNA profiling, together with genomics and proteomics, could lead to unraveling regulatory networks of biological processes related to cancer^10^.

In this study, we used high-throughput proteomics and microRNA profiling to characterize two subtypes of breast cancer with various prognoses: ER+/progesterone receptor-positive (PR+) HER2- breast cancer and triple-negative breast cancer (TNBC). We applied probabilistic graphical models and flux balance analyses to explore molecular differences between these subtypes to unveil differences not detected by immunohistochemistry or genomics.

## Results

### Patient characteristics

A total of 106 patients with breast cancer from two different hospitals were included in the discovery cohort. All the patients had node-positive disease, all the tumors were negative for HER2 and all had received adjuvant chemotherapy and hormonal therapy for patients with ER+ disease (patients showing estrogen and/or progesterone receptor expression). Forty-six additional patients from a third hospital with ER+ disease and nodal involvement were eligible for the verification cohort: all had received anthracycline-based adjuvant chemotherapy followed by hormone therapy (Table 1 and Sup. Fig. S1).

**Table 1:** Patient’s characteristics.

	All	Discovery	ER+	Verification
		TNBC		All
Number of patients	106	26	80	46
Age at diagnosis (median)	54.6 (32–83)	61.2 (37–78)	54.2 (32–83)	55 (39–70)
Age at diagnosis (mean)	55.2	58.5	54.1	53.9
**Tumor Size**				
T1	33 (31%)	5 (19%)	28 (35%)	19 (41%)
T2	61 (58%)	19 (73%)	42 (53%)	21 (46%)
T3	10 (9%)	2 (8%)	8 (10%)	6 (13%)
T4	1 (1%)	0 (0%)	1 (1%)	0 (0%)
Multifocal	1 (1%)	0 (0%)	1 (1%)	0 (0%)
**Tumor Grade**				
G1	12 (11%)	0 (0%)	12 (15%)	6 (13%)
G2	33 (31%)	4 (15%)	29 (36%)	22 (48%)
G3	41 (39%)	20 (77%)	21 (26%)	12 (26%)
Unknown	20 (19%)	2 (8%)	18 (23%)	6 (13%)
**Lymph node status**				
N0	0 (0%)	0 (0%)	0 (0%)	0 (0%)
N1	71 (67%)	17 (65%)	54 (68%)	39 (85%)
N2	35 (33%)	9 (35%)	26 (32%)	7 (15%)
**Chemotherapy**				
No anthracyclines	34 (32%)	11 (42%)	23 (29%)	0 (0%)
Anthracyclines	63 (59%)	12 (46%)	51 (64%)	66 (100%)
Anthracyclines + taxanes	9 (9%)	3 (12%)	6 (7%)	0 (0%)

### Protein extraction and shotgun-mass spectrometry analyses of formalin-fixed, paraffin-embedded breast cancer tumors

After mass spectrometry (MS) workflow, 25 TNBCs and 71 ER+ tumors from the discovery cohort were analyzed. Raw data normalization was performed as previously described^10^. Four samples were excluded due to poor protein extraction and six were excluded due to data quality. Of 3,239 protein groups identified using Andromeda, 1095 presented at least two unique peptides and detectable expression in at least 75% of the samples in either the ER+ or TNBC groups. No decoy protein passed through these additional filters. Label-free quantification data were obtained using MaxQuant as previously described^10^.

### Protein expression analyses of breast cancer tumors

**Figure 1:**
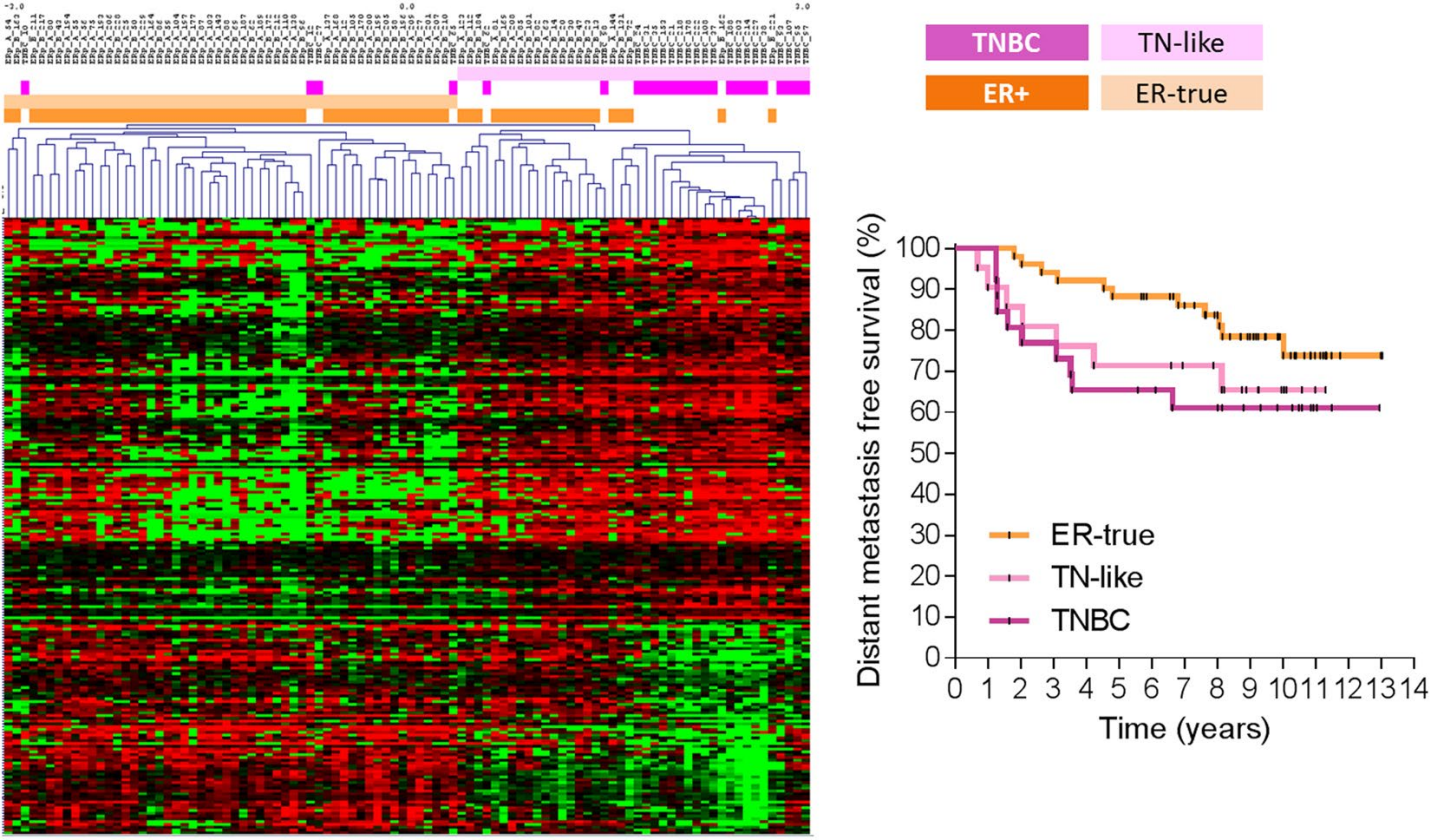
ER-true/TN-like subtype definition and characterization. Left panel: Hierarchical clustering analysis from 224 proteins identified by SAM analysis between ER+ and TNBC tumors with FDR < 5%. Right panel: Kaplan-Meier analysis showing survival for ER-true, TN-like and TNBC tumors (n = 51, 21 and 26, respectively; p = 0.17).

Protein expression values were analyzed using Significance Analysis of Microarrays (SAM). A total of 224 proteins were differentially expressed between the ER+ and TNBC samples with a false discovery rate (FDR) <5% (Sup. Table S1). Hierarchical clustering analysis split the samples into two main clusters: cluster I comprised 70.4% of ER+ tumors (labeled ER-true), and cluster II included both ER+ and triple-negative (TN) tumors. The ER+ tumors included in cluster II, representing 29.6% of all ER+ tumors, were labeled as TN-like tumors. The distant metastasis-free survival (DMFS) rate at 5 years was 88.2% for ER-true and 71.4% for TN-like patients (p = 0.21). The clinical evolution of TN-like breast cancer was similar to that of TNBC (DMFS rate at 5 years 65.4%, p = 0.7) (Fig. 1).

### Characterization of ER-true and TN-like subtypes

A significance analysis of microarrays (SAM), excluding TNBC tumors, was performed to further characterize ER-true and TN-like subtypes. We found 44 proteins showing differential expression between both subgroups, with an FDR < 5% (Sup. Table S2 and Sup. Fig. 2). Four proteins presented deleted records in Uniprot and were excluded. Among the proteins with higher expression in ER-true tumors, we found 7 extracellular small leucine-rich canonical proteoglycans (SLRPs) (biglycan, decorin, asporin, lumican, prolargin, fibromodulin and osteoglycin), three proteins produced by mast cells (cathepsin G, mast cell carboxypeptidase A and chymase), COEA1, PRDBP, and both the PIP and ZA2G proteins. On the other hand, TN-like tumors showed greater expression of HS90B and STIP1 from the chaperone pathway, EF2 and THEM6 proteins. Gene ontology analyses showed that proteins defining the TN-like subtype were related to cell adhesion processes (Sup. Table S3). Regarding clinical factors, we found that TN-like tumors showed higher molecular grade (G1-2 vs. G3, p = 0.03). No differences between ER-true and TN-like tumors regarding age at diagnosis, tumor size, number of affected nodes, and ER, PR or Ki67 pathological assessment were found.

### MicroRNA expression analysis of breast cancer tumors

MicroRNA expression profiling was available for 42 ER-true and 23 TN-like tumors from the discovery cohort. One microRNA was excluded from subsequent analyses due to absence of expression in most of the samples. Nine microRNAs showed significant higher expression in the ER-true compared with the TN-like tumors (p < 0.05; FDR < 5%) (Sup. Fig. S3).

### Systems biology of ER+ breast cancer

**Figure 2:**
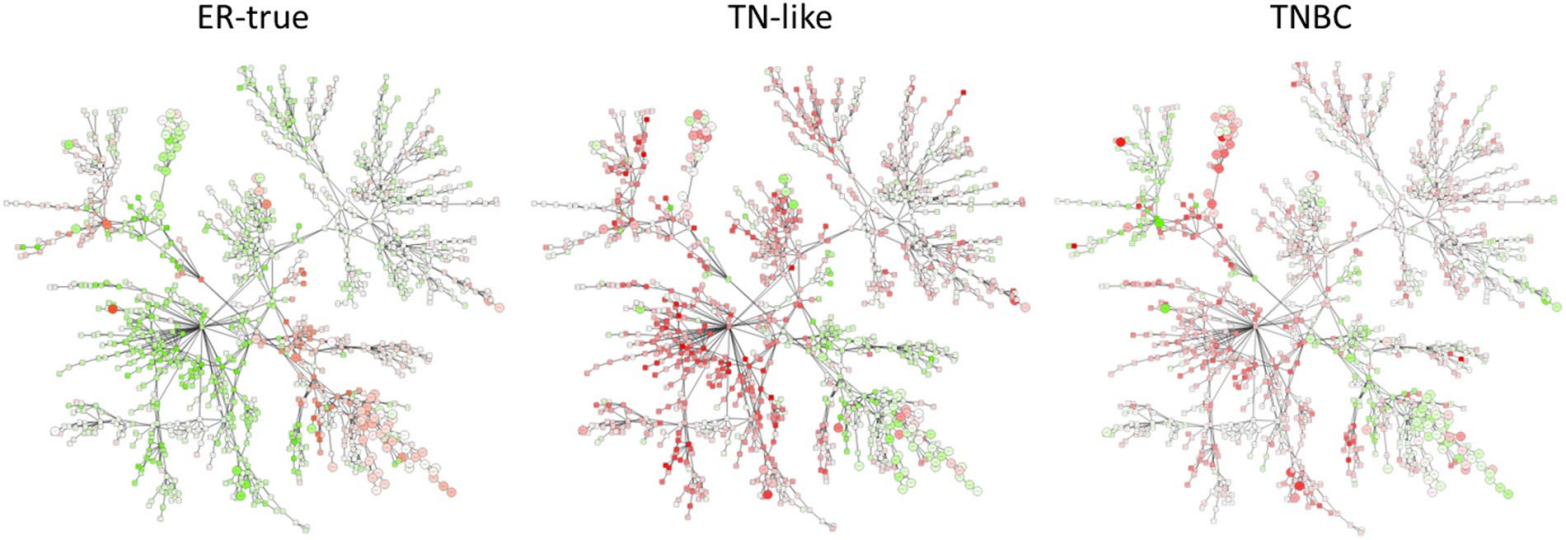
Protein- and miRNA-based probabilistic graphical model. Probabilistic graphical model showing protein (squares) and miRNA (circles) mean expression in each sample type. Color range from -2-fold change (green) to 2-fold change (red). White means no change between groups. ER-true subtype is compared with TN-like subtype and *vice versa*. TNBC type is compared with all ER+ tumors.

Both label-free protein quantification and microRNA expression data were available for 16 TNBC and 63 ER+ tumors from the discovery cohort. A probabilistic graphical model was constructed with these values as previously described^10^. Differences in functional node activity between ER-true and TN-like tumors were found (Figs 2 and S4). These differences were corroborated in the external dataset (p < 0.05), except for the protein synthesis node. All metabolism and mitochondria nodes present higher activity in TN-like tumors. The “metabolism A” node includes proteins related to glutamine and glucose metabolism and LDHB. The “metabolism B” node includes GAPDH, PGK1, LDHA and pyruvate kinase proteins, among others; and also miR-449a, whose expression showed a negative correlation with the functional node activity (Sup. Fig. S6). The “mitochondria A” node includes proteins related to the mitochondrial oxidation/reduction process, whereas the “mitochondria B” node comprises tricarboxylic acid (TCA) cycle proteins. The “ECM & focal adhesion” node showed higher activity in ER-true tumors, and includes miR-139-5p, miR-149, miR-766, miR-342, miR-214* and miR-31. Both miR-214* and miR-31 expression showed positive correlation with functional node activity (Sup. Fig. S5). The “response and membrane” node includes proteins related to cellular response to external stimuli and cholesterol homeostasis, and shows higher activity in ER-true tumors. The “proteasome” node includes proteins from the proteasome core complex, and showed higher activity in TN-like tumors. This functional node includes miR-489 and miR-99a, although no correlation was found between their expression and functional node activity.

### Flux balance analysis of breast tumors

**Figure 3:**
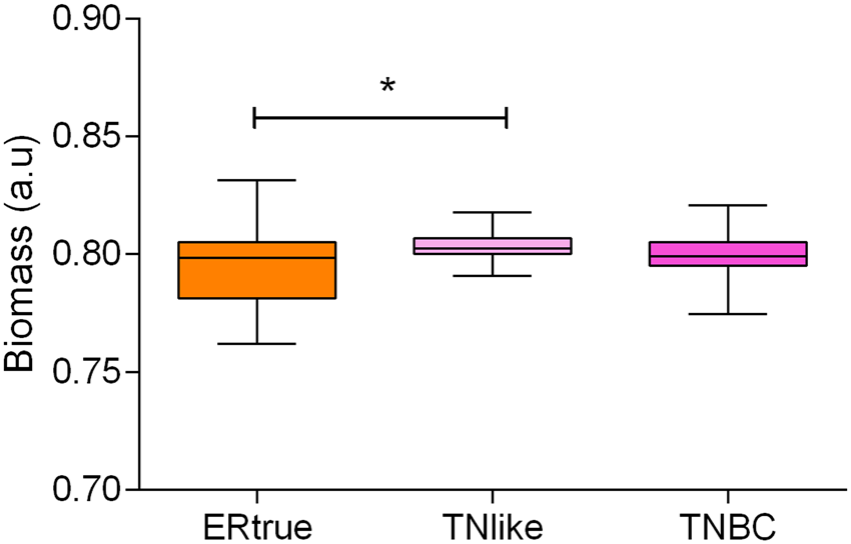
Tumor growth rate predicted by flux balance analysis. FBA results for ER-true, TN-like and TNBC tumors (n = 51, 21 and 26, respectively; *p < 0.05).

We performed a flux balance analysis (FBA) using the E-Flux algorithm to evaluate the impact of the proteomics profile on tumor growth capability^11^. Our Recon 2-based model includes 7440 reactions, from which we found gene-protein-reaction (GPR) rule values mediating 1085 reactions. All the tumors fulfilled the Warburg effect, redirecting pyruvate generated by glycolysis and glutaminolysis to lactic fermentation through lactate dehydrogenase. The predicted tumor growth rate was higher in both the TN-like and TNBC tumors compared with the ER-true tumors (Fig. 3).

### Targeted proteomics of TN-like/ER-true subtypes

**Figure 4:**
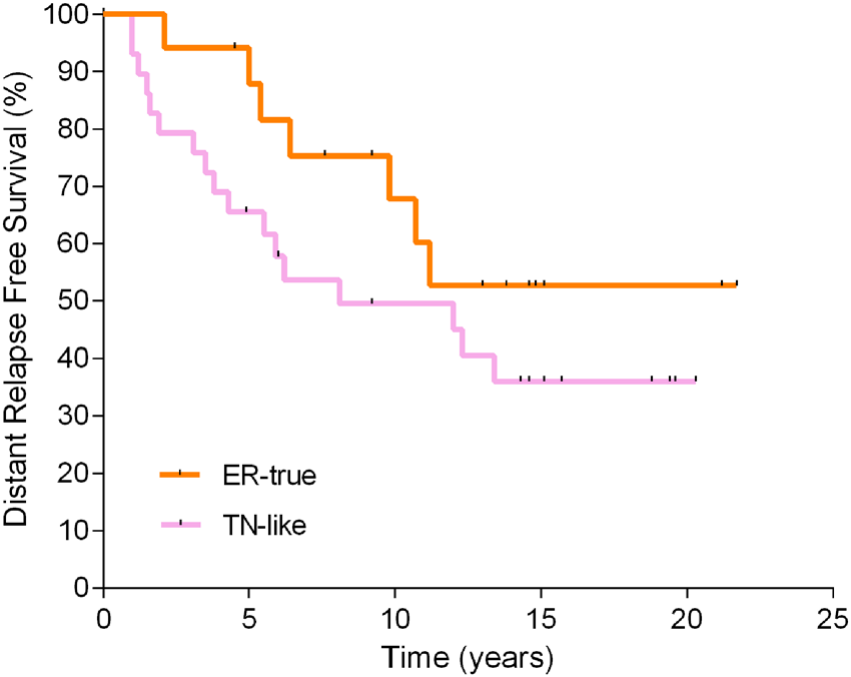
SRM validation of new subtypes. Kaplan-Meier analysis showing survival rates for ER-true and TN-like tumors on the basis of SRM data (n = 17 and 29, respectively).

To corroborate the prognostic value of the TN-like/ER-true classification, 33 proteins differentially expressed between TN-like and ER-true subtypes were assessed, using a targeted proteomics approach via selected reaction monitoring (SRM) in a new cohort comprising 46 ER+ breast cancer tumors (Table 1)^12^. One sample was excluded due to poor protein extraction and two due to data quality. Nineteen samples from the discovery cohort were also tested. SRM was able to detect differences between ER-true and TN-like samples from the discovery cohort (Sup. Fig. S6). An ER-true/TN-like classifier, including 14 proteins, was used to assign new samples to ER-true or TN-like (sup. info). DMFS rates at 5 years were 81.6% and 57.8% for the ER-true and TN-like groups, respectively (p < 0.17) (Fig. 4).

### Assessing ER-true/TN-like subtypes using a meta-genomics external dataset

We used gene expression data from 1296 breast cancer tumors, obtained from public repositories, as an independent cohort to validate the prognostic value of the ER-true/TN-like stratification^13, 14^. Among them, 935 tumors were ER+ and had follow-up information available. Tumors were labeled as ER-true or TN-like using 35 of 44 proteins from SAM analyses. Survival analyses using 421 tumors with ER+ and node positive characteristics showed that DMFS rates were 81.8% and 72.5% for the ER-true and TN-like groups, respectively (p < 0.005, HR = 0.5769, Sup. Fig. S7).

### ER-true/TN-like subtypes and breast cancer molecular subtypes

**Figure 5:**
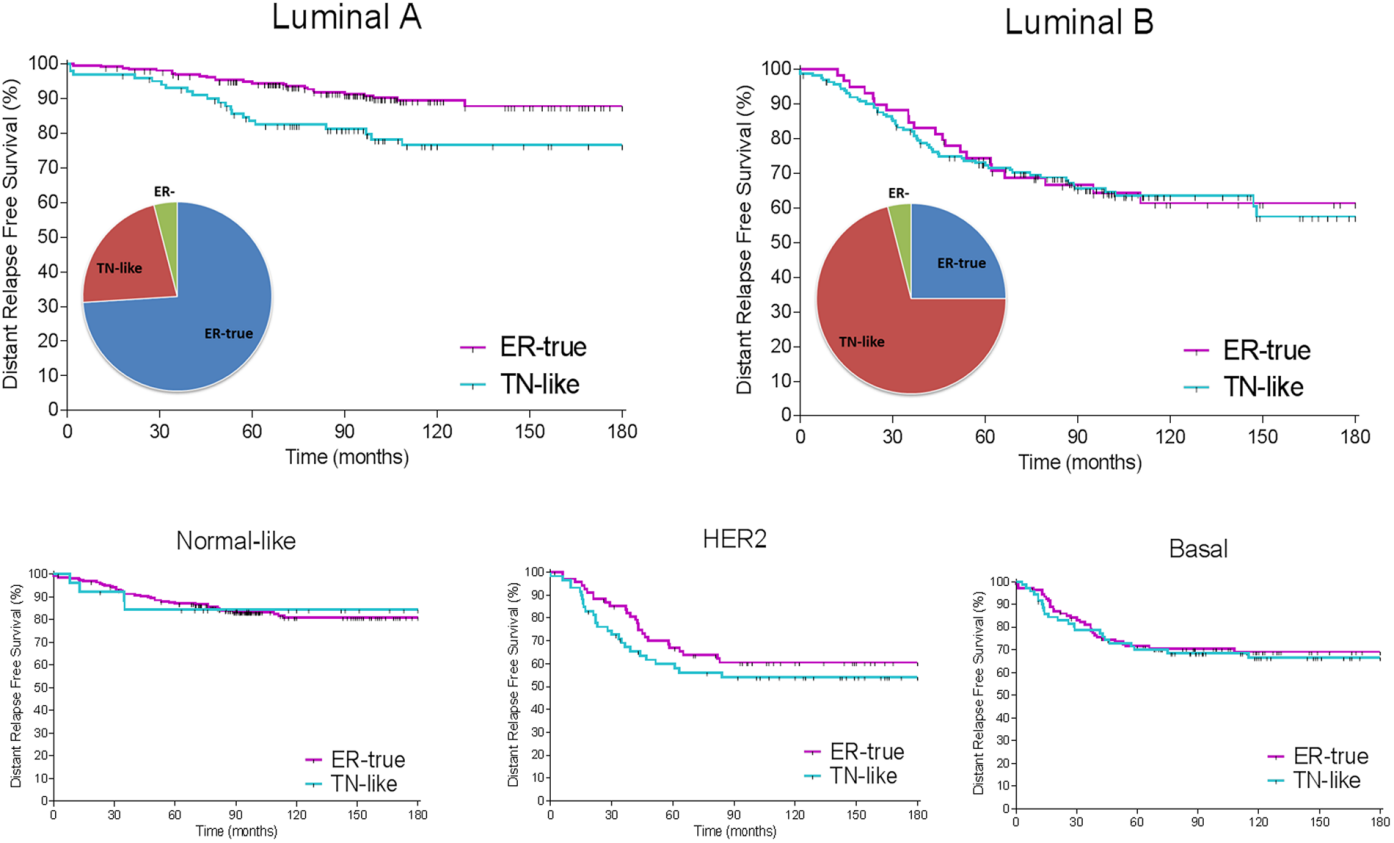
Prognostic value of ER-true/TN-like subtype within breast cancer molecular subtypes. Kaplan-Meier analysis showing ER-true and TN-like tumor survival rates in luminal (**A**) (left panel: ER-true n = 262, TN-like n = 101) and luminal (**B**) (right panel: ER-true n = 59, TN-like n = 164) subtypes.

We applied our TN-like classifier to the entire population and performed survival analyses independently for each breast cancer molecular subtype^3^. ER-true/TN-like subtyping provided additional prognostic information in luminal A tumors, but not in luminal B, basal or HER2-enriched tumor subtypes (Fig. 5).

### ER-true/TN-like subtypes and molecular prognostic signatures

**Figure 6:**
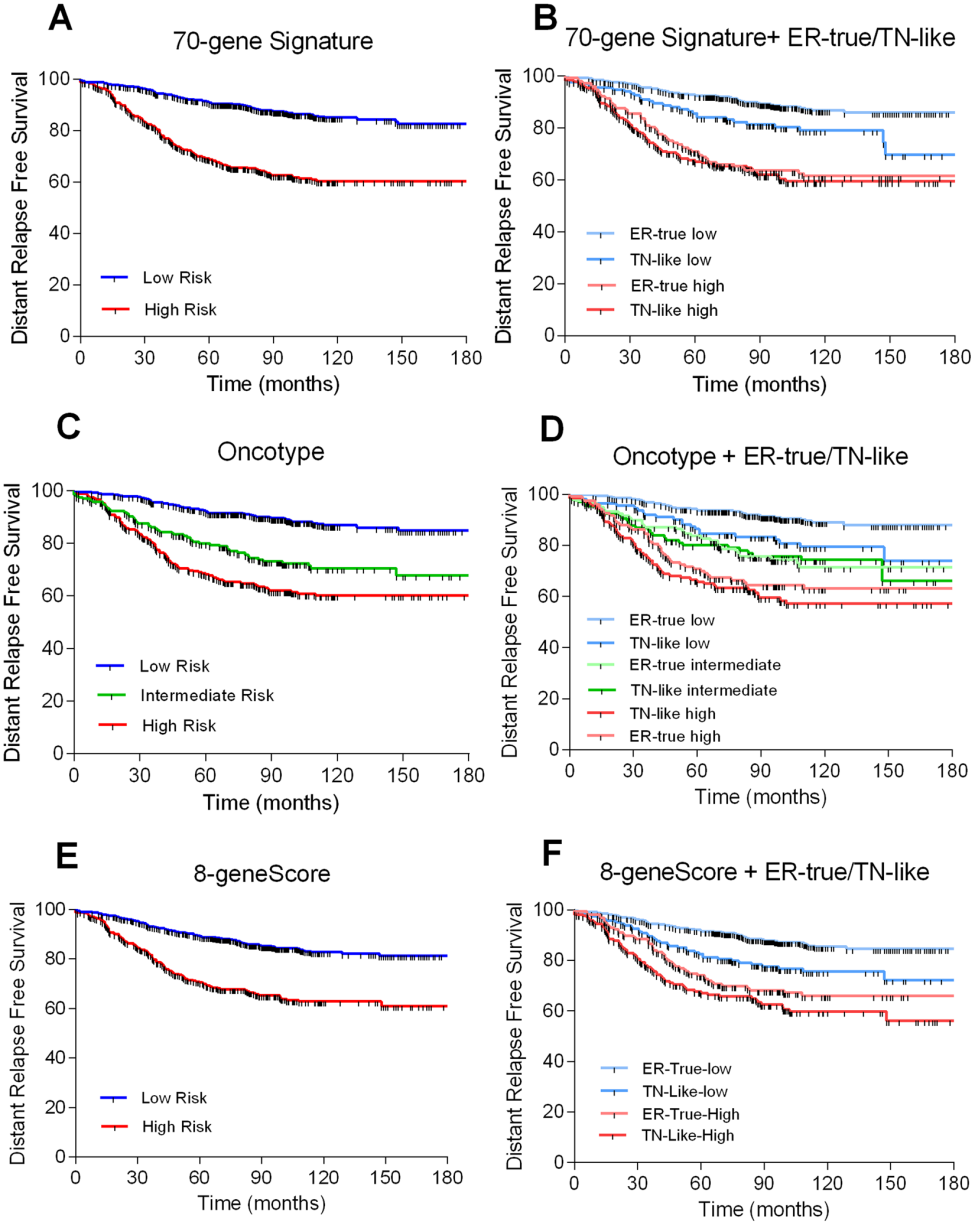
ER-true/TN-like subtype and prognostic signatures. Kaplan-Meier analysis showing survival rates of risk groups defined by prognostic gene signatures and ER-true/TN-like subtypes. (**A**) 70-gene Signature: Low risk = 586; High risk = 349; p < 0.0001; HR = 3.24 (2.73–4.85). (**B**) 70-gene Signature and ER-true/TN-like subtypes: Low risk/ER-true = 449; High risk/ER-true = 154; Low risk/TN-like = 137; High risk/TN-like = 195; p < 0.0001. (**C**) Recurrence Score: Low risk = 472; Intermediate risk = 195; High risk = 268; p < 0.0001. (**D**) Recurrence Score and ER-true/TN-like subtypes: Low risk/ER-true = 358; Intermediate risk/ER-true = 120; High risk/ER-true = 268; Low risk/TN-like = 125; Intermediate risk/TN-like = 108; High risk/TN-like = 143; p < 0.0001. (**E**) 8-gene Score: Low risk = 610; High risk = 325; p < 0.0001; HR = 2.61 (2.19–3.94). (**F**) 8-gene Score and ER-true/TN-like subtypes: Low risk/ER-true = 445; High risk/ER-true = 158; Low risk/TN-like = 165; High risk/TN-like = 167; p < 0.0001.

The clinical utility of the ER-true/TN-like subtypes was evaluated in combination with three prognostic gene signatures: the 70-gene signature^15^, the Recurrence Score^16^ and the 8-gene Score^17^. The prognostic value of the three tests in 935 patients with ER+ tumors was corroborated, followed by the application of the ER-true/TN-like class predictor (Fig. 6). The TN-like tumors were associated with a lower DMFS compared with ER-true tumors in each low-risk category, defined by prognostic signatures (Sup. Table S4). The high-risk categories were not further refined through ER-true/TN-like subtyping. The multivariate analyses, including each prognostic signature and the ER-true and TN-like subtypes, showed that the ER-true and TN-like subtypes were related to prognosis, independent of the prognostic gene signatures (Table 2). Multivariate analyses including the ER-true/TN-like subtypes and available clinical variables (grade and N) showed that both grade and ER-true/TN-like subtypes, along with lymph node status, provided significant and independent prognostic information.

**Table 2:** Univariate and multivariate analyses including clinical variables, prognostic signatures and the TN-like subtype.

Univariate analysis		
	p-value	HR
ER-true/TN-like subtype	<10^−4^	1.911
70-gene Signature	<10^−4^	3.239
Recurrence Score	<10^−4^	1.929
8-gene Score	<10^−4^	2.605
**Multivariate analysis TN-like subtype and clinical variables**		
ER-true/TN-like subtype	0.022	1.374
Grade (1 + 2 vs. 3)	>10^−4^	1.555
N	0.005	1.481
**Multivariate analysis TN-like subtype and prognostic signatures**		
ER-true/TN-like subtype	0.05	1.329
70-gene Signature	>10^−4^	2.948
ER-true/TN-like subtype	0.011	1.441
Recurrence Score	>10^−4^	1.829
ER-true/TN-like subtype	0.002	1.544
8-gene Score	>10^−4^	2.336

## Discussion

In this study, a new subtype of breast cancer was identified using a proteomics approach. The clinical classification of breast cancer does not fully reflect cancer heterogeneity; thus, individuals receiving the same diagnosis can have markedly different outcomes. Genomics and proteomics approaches complement the information provided by routine determinations, and coupled with new data analysis techniques, they help to expand the information obtained. In this case, information provided by pure protein expression was organized into functional nodes involving specific biological processes and pathways. The new TN-like ER+ subtype defined has molecular features common with TNBC tumors and exhibits a similar clinical evolution. Patients with either TN-like or TNBC tumors have shorter DMFS than patients with ER-true breast cancer. Both SRM verification and meta-validation confirmed the findings obtained in the discovery series. These results might help to explain why the prognosis of patients with ER+ breast cancer is not uniformly favorable.

ER-true tumors present molecular features that could explain the favorable prognosis of this subtype, such as increased expression of proteins related to cell adhesion and greater activity of the “ECM & focal adhesion” node. Increased expression of decorin and lumican in breast cancer is associated with lower tumor size, decreased risk and rate of relapse, positive ER/PR status and better survival^18, 19^. A stromal gene set including DCN and FBLN1 genes has demonstrated prognostic value independent of clinical information and a proliferation gene set^20^. COEA1, asporin, osteoglycin and lumican showed increased expression in low-risk vs. high-risk tumors defined by MammaPrint^21^. With regard to miRNAs included in the “ECM & focal adhesion” node, miR-342 expression correlates with ER expression and tamoxifen sensitivity in breast tumors^22–24^. Both miR-149 and miR-342 have been included in a prognostic signature for breast cancer^25^. Our results suggest that miR-31 and miR-214* could be indirect regulators of cell attachment function in breast tumors. These results indicate that ER-true tumors harbor a limited metastatic potential compared with TN-like tumors.

There is more limited information on some other molecular features defining ER-true tumors. This subtype has high expression for proteins produced by mast cells, related to ER and PR positivity, low-grade and a good prognosis in breast cancer^26–29^. High expression levels of PIP and AZGP1 genes have been related to a good prognosis and correlate with ER, PR and AR expression^21, 30–38^. ZA2G is part of a panel of 13 proteins predicting recurrence in breast cancer, showing decreased expressions when recurrence occurred^39^. PRDBP protein appears to dictate the balance between ERK and Akt signaling with consequences for cell metabolism (induction of Warburg metabolism), apoptosis and cell proliferation^40^. Loss of the 11p15 region, where the PRKCDBP gene is located, is common in breast cancer metastases^41^.

TN-like tumors showed molecular features associated with an unfavorable prognosis. High HSP90AB1 expression is related to poor overall survival and with an increased distant metastasis relapse rate in breast cancer^42, 43^ which is consistent with our results, showing a higher expression of this protein in TN-like tumors. HS90B has been included in a panel of 13 proteins predicting recurrence in breast cancer^39^. STIP1 interacts with HS90B in the folding of a number of proteins, including the androgen and estrogen receptors^44, 45^. Additionally, greater expression of eEF2 was significantly associated with node positivity in breast cancer^46^.

On the other hand, all metabolism and mitochondria nodes had higher activity in the TN-like subtype. The “mitochondria B” and “metabolism B” nodes include proteins related to the TCA cycle and glycolysis, respectively, suggesting that both TN-like and TNBC tumors are highly glycolytic. The TN-like tumors showed high activity in both the “metabolism A” and “mitochondria A” nodes compared with the ER-true and TNBC tumors, suggesting a unique metabolic profile for the TN-like subtype. FBA indicates that all breast cancer types fulfill the Warburg hypothesis and that glutamine-derived αKG refuels the TCA cycle (anaplerosis) and maintains constant levels of biosynthetic precursors, while the surplus turns to lactate^47^. However, ER-true tumors had a predicted growth rate significantly lower than TN-like and TNBC tumors, both of which had comparable growth rates.

Molecular differences between TN-like and ER-true tumors resemble those previously described between ER+ and TNBC tumors^10^. SAM analysis identified 44 proteins differentially expressed between both subtypes, 24 of which were also differentially expressed between ER+ and TNBC samples. Moreover, miR-139-5p, miR-149, miR-449a and miR-342 were overexpressed in ER+ tumors with regard to TNBCs^10, 24, 48, 49^. Interestingly, we found equivalent differences in the “ECM & focal adhesion,” “metabolism B,” “mitochondria B” and “protein synthesis” nodes when comparing ER-true versus TN-like tumors and ER+ versus TNBC tumors. Differences in the “protein synthesis” node could not be confirmed in the external dataset in both analyses, suggesting that some features observed at the protein level do not appear at the gene expression level^10^. On the other hand, no differences regarding the “proliferation” node activity between ER-true and TN-like samples were found, although they were present between the ER+ and TNBC tumors. We also found differences not described between ER+ and TNBC tumors: the “mRNA processing” and “protein transport” nodes showed higher activity in ER-true tumors, whereas the “response and membrane” node had higher activity in TN-like tumors.

The TN-like subtype added prognostic information in luminal A disease but not in the other molecular subtypes. Likewise, the TN-like subtype further subdivided low-risk categories defined by gene signatures, such as the 70-gene Score, Recurrence Score and 8-gene Score. These gene signatures are related to cell proliferation, whereas the TN-like subtype primarily depends on other drivers, such as cell attachment and metabolism, thus providing complementary information^50^. New molecular information could improve the accuracy of gene signatures and help to determine the best treatment for patients with ER+ breast cancer. Additionally, the TN-like subtype prognostic information is independent of that provided by clinical variables such as lymph node status and grade. Adjuvant treatment of breast cancer is determined by two main factors: risk of relapse and the molecular characteristics of the tumor. Molecular tools developed in this setting — such as MammaPrint, OncoType or the 8-gene Score — have attempted to optimize the use of adjuvant chemotherapy, which is toxic and benefits a limited number of patients. Patients in the low-risk categories of these gene tests do not require chemotherapy, but our results indicate that these low-risk categories can be further subdivided. The presence of a TN-like subtype worsens the outcome; therefore, chemotherapy should be considered in these patients. The recommendation would be valid for luminal A tumors having features of the TN-like subtype, which could contribute to reducing the number of relapses in this population.

The TN-like subtype provided prognostic information in ER+ disease not only with the original proteomics approach, but also with other techniques, including the translation of proteins back to gene expression. This result supports the robustness of this new breast cancer subtype. In addition, some of the components defining the subtype could become potential therapeutic targets in the future. Hormonal receptors and HER2 are the only molecular features allowing targeted therapy in breast cancer. Gene subtyping into luminal A, luminal B, basal and HER-2 enriched groups has not revealed other features that can be used to develop new drugs. A proteomics approach unravels molecular processes not detected by genomics, with the advantage that proteins are the real effectors of genomic changes.

Our study has some limitations. The discovery series was limited to patients with node-positive disease, who have a poorer outcome than their node-negative counterparts. However, the meta-validation series is more heterogeneous regarding clinical stage, which suggests that the TN-like subtype is a clinical entity and not just a marker of advanced disease. Also, relevant clinical differences in the discovery and verification cohorts did not reach the statistical boundary due to the limited sample size and the fact that many relapses in this group appeared after 5 years of follow-up. This problem was overcome in the *in-silico* series, which is more representative of a population of patients with breast cancer. On the technical side, despite the informative value of proteomics, there is still room for improvement in the number of proteins detected. Moreover, SRM assays are complex to develop and analyze in comparison with other platforms such as quantitative polymerase chain reaction (qPCR), and its use in the clinical routine is still challenging. Finally, these results should be validated in additional cohorts to evaluate the TN-like subtype robustness.

High-throughput proteomics generate clinically useful protein-based molecular profiles, which can complement information provided by gene expression analysis. In this study, a proteomics approach allowed the identification of a new subtype of breast cancer using FFPE samples. The molecular characteristics of this new subgroup have been assessed using probabilistic graphical models. This subtype is included in the group of hormonal receptor-positive, HER2-negative tumors, but has molecular features and a poor clinical outcome similar to that of TNBC. This new TN-like subtype has the capability to add prognostic information to current clinical practice. Because proteins are the final effectors of genes, some proteins and biological processes defining TN-like tumors could become therapeutic targets. This possibility should be further explored in future studies.

## Methods

### Sample selection

A total of 106 patients with breast cancer were included in the discovery cohort. FFPE samples were retrieved from the I+12 Biobank (RD09/0076/00118) and from the IdiPAZ Biobank (RD09/0076/00073), both integrated in the Spanish Hospital Biobank Network (RetBioH; www.redbiobancos.es). Forty-six patients were included in the verification cohort, and FFPE samples were retrieved from the Basque Biobank/O+EHUN (RD09/0076/00140). Informed consent was obtained from all the patients. All the experiments were performed in accordance with relevant guidelines and regulations. The histopathological features of each sample were reviewed by an experienced pathologist to confirm diagnosis and tumor content. Eligible samples included at least 50% tumor cells. Approvals from the Ethics Committees of Hospital Doce de Octubre, La Paz University Hospital and Euskadi were obtained for the conduct of the study.

### Total protein preparation and digestion

Proteins were extracted from FFPE samples as previously described^51^. Briefly, FFPE sections were deparaffinized in xylene and washed twice with absolute ethanol. Protein extracts from the FFPE samples were prepared in 2% sodium dodecyl sulfate (SDS) buffer using a protocol based on heat-induced antigen retrieval^52^. Protein concentration was determined using the MicroBCA Protein Assay Kit (Pierce-Thermo Scientific). Protein extracts (10 µg) were digested with trypsin (1:50) and SDS was removed from digested lysates using Detergent Removal Spin Columns (Pierce). Peptide samples were further desalted using ZipTips (Millipore), dried, and resolubilized in 15 µL of a 0.1% formic acid and 3% acetonitrile solution before MS analysis.

### Liquid chromatography - mass spectrometry shotgun analysis

The samples were analyzed on an LTQ-Orbitrap Velos hybrid mass spectrometer (Thermo Fischer Scientific, Bremen, Germany) coupled to a NanoLC-Ultra system (Eksigent Technologies, Dublin, CA, USA) as previously described previously^10^. Briefly, after separation, peptides were eluted with a gradient of 5% to 30% acetonitrile in 95 minutes. The mass spectrometer was operated in data-dependent mode (DDA), acquiring a full-scan MS spectra (300–1700 m/z) at a resolution of 30,000 at 400 m/z after accumulation to a target value of 1,000,000, followed by collision-induced dissociation (CID) fragmentation on the 20 most intense signals per cycle. The samples were acquired using internal lock mass calibration on m/z 429.088735 and 445.120025. The acquired raw MS data were processed by MaxQuant (version 1.2.7.4)^53^, followed by protein identification using the integrated Andromeda search engine^54^. Briefly, spectra were searched against a forward UniProtKB/Swiss-Prot database for human, concatenated to a reversed decoyed FASTA database (NCBI taxonomy ID 9606, release date 2011-12-13). The maximum FDR was set to 0.01 for peptides and 0.05 for proteins. Label-free quantification was calculated on the basis of the normalized intensities (LFQ intensity). Quantifiable proteins were defined as those detected in at least 75% of samples in at least one type of sample (either ER+ or TNBC samples) showing two or more unique peptides. Only quantifiable proteins were considered for subsequent analyses. Protein expression data were log2 transformed, and missing values were replaced using data imputation for label-free data, as explained in Deeb *et al*.^55^, using default values. Finally, protein expression values were z-score transformed. Batch effects were estimated and corrected using ComBat^56^. All the mass spectrometry raw data files acquired in this study can be downloaded from Chorus (http://chorusproject.org) under the project name *Breast Cancer Proteomics*.

### RNA extraction and MicroRNA expression

RNA isolation from the FFPE tumor specimens and microRNA expression profiling was performed as previously described^10^. Briefly, microRNA expression profiling was obtained using a custom TaqMan Array MicroRNA Card (Applied Biosystems) containing 95 FFPE-reliable assays, including four housekeeping miRNAs identified used NorMean^57^. ΔCq values were normalized using two reference miRNAs (hsa-let-7d and hsa-let-7g).

### Differential expression analysis of label-free proteomics and microRNA profiling

SAM^58^ was performed to find differentially expressed proteins and miRNAs between sample groups with an FDR below 5%. Hierarchical clusters were constructed with the differentially expressed proteins or miRNAs between predefined samples groups identified by SAM, using Pearson’s correlation and the average-linkage method.

### Functional network construction

A functional network to associate miRNAs and protein expression profiles was constructed as previously described^10^. Briefly, we chose probabilistic graphical models compatible with high-dimensionality. The result is an undirected graphical model with a local minimum Bayesian Information Criterion (BIC)^59^. Methods are implemented in the open-source statistical programming language R^60^; in particular, the functions *minForest* and *stepw* in the *gRapHD* package^61^. To identify functional nodes within the network, we split it into several branches or functional nodes. We then used gene ontology analyses to investigate which function or functions were overrepresented in each branch. To measure the functional activity of each functional node, we calculated the mean expression of all the proteins included in one branch related to a specific function. Differences in functional node activity were assessed by class comparison analyses.

### Gene ontology analyses

Protein-to-gene ID conversion was performed using Uniprot (http://www.uniprot.org) and DAVID^62, 63^. Gene ontology analyses were performed using the functional annotation chart tool provided by DAVID. We used “*homo sapiens*” as a background list and selected only GOTERM-FAT gene ontology categories and Biocarta, KEGG and Panther pathways.

### Flux balance analyses

Flux balance analysis (FBA) is a widely used approach for studying biochemical networks by calculating the flow of metabolites through the network, including 7440 reactions from Recon 2^64^. With this method, it is possible to predict the growth rate of an organism or the rate of production of a metabolite^65^. The estimation of the GPR rule values was performed using a variation of the method described by Barker *et al*.^66^. The mathematical operations used to calculate the numerical value were the sums for “OR” expressions and minimums for “AND” expressions. Finally, the GPR rule values were normalized, dividing by the maximum value in each tumor, and were included in the Recon 2 model using the E-Flux algorithm^11^. Normalized GPR rule values have been used to establish both lower and upper reaction bounds if the reaction is reversible. If the reaction is irreversible, low bound is set to 0 in all cases. To calculate biomass production, the biomass objective function included in Recon 2 was optimized. FBA was performed using the COBRA Toolbox available for MATLAB^67^.

### Selected reaction monitoring analyses

The SRM design was based on both experimental data from our shotgun analysis and the PeptideAtlas^68^. SRM-triggered MS2 was performed on a QTRAP 5500 instrument (ABSciex, Concord, Ontario), and SRM measurements were analyzed on a TSQ Vantage Triple Quadrupole Mass Spectrometer (ThermoFisher, San Jose, CA, USA), both equipped with a nanoelectrospray ion source. Chromatographic separations of peptides were performed on a NanoLC-2D HPLC system (Eksigent, Dublin, CA) coupled to a 15-cm fused silica emitter, 75-μm diameter, packed with a ReproSil-Pur C18-AQ 120 A and 1.9-μm resin (Dr. Maisch HPLC GmbH). Peptides were loaded on the column from a cooled (4 °C) Eksigent autosampler and separated with a linear gradient of acetonitrile/water, containing 0.1% formic acid, at a flow rate of 300 nl/min. A gradient from 5% to 35% acetonitrile in 40 minutes was used. For the SRM-triggered MS2 measurements, MS2 spectra were recorded upon detection of an SRM trace above a threshold of 1000 ion counts. An average of 100 transitions (scan time 10 ms/transition) per run was used and Q1 and Q3 were obtained at 0.7 unit mass resolution. MS2 spectra were recorded in enhanced product ion (EPI) mode for the highest MRM transitions, using dynamic fill time, Q1 resolution unit, scan speed 10,000 amu/s, m/z range 300–1000. Collision energies used for both acquisition modes were calculated according to the formulas: CE = 0.044 * m/z + 5.5 and CE = 0.051 * m/z + 4 (CE: collision energy; m/z: mass-to-charge ratio of the precursor ion) for doubly and triply charged precursor ions, respectively. In SRM, the mass spectrometer was operated in SRM scan mode, in which Q1 and Q3 were obtained at 0.7 unit mass resolution. Collision energies for each transition were calculated according to the following equations: CE = 0.034 * (m/z) + 3.314 and CE = 0.044 * (m/z) + 3.314 for doubly and triply charged precursor ions, respectively. Three SRM transitions were monitored for each endogenous (light) and internal standard (heavy) peptide. SRM data were processed using SRM skyline software^69^. Peptides with the following criteria were used for the quantification: (i) correlation between ion ratios obtained for the heavy and the light form; (ii) correlation between the ion ratios obtained for both forms and the ion ratios obtained in the MS/MS spectra present in the SRM spectral library; and (iii) transition intensities of the heavy and the light form of >10. The three transitions for each heavy-light pair were used to quantify the peptide unless signals of coeluting interferences were detected. Punctual measurements for light peptides below the background measurement value were ignored. A light/heavy peptide ratio was calculated for all transitions. Protein expression values were calculated by the median expression from the three transitions for each heavy-light pair of their peptides.

### Development of classifiers

We developed protein expression-based signatures to predict the class of future samples using the compound covariate predictor. The model incorporates proteins that were differentially expressed among classes at the 0.05 significance level as assessed by the random variance t-test^70^, with protein-to-gene ID positive in the meta-validation dataset (see below). We estimated the prediction error of each model using leave-one-out cross-validation (LOOCV)^71^. For each LOOCV training set, the entire model building process was repeated, including the gene selection process. We also evaluated whether the cross-validated error rate estimate for a model is significantly less than the random prediction. The class labels were randomly permuted and the entire LOOCV process was repeated. The significance level is the proportion of the random permutations that gave a cross-validated error rate no greater than the cross-validated error rate obtained with the original data. The same workflow was performed using the SRM data. For more details, see the Simon R and Lam A. BRB-ArrayTools User Guide, version 3.2. BRB-ArrayTools v4.2.1, developed by R. Simon and A. Peng.

### External dataset validation

A total of 1296 primary breast carcinoma data were collected from two independent datasets^13, 14^. The Guedj dataset and associated clinical annotations were downloaded from the ArrayExpress Archive (http://www.ebi.ac.uk/arrayexpress/). The Miller dataset and associated clinical annotations were downloaded from the Cancer Research website. Batch effects were corrected using ComBat^56^. Protein-to-gene ID was performed using Uniprot (http://www.uniprot.org) and DAVID^62, 63^. All the probes in the dataset for each gene were retrieved. Probes with higher coefficients of variation were selected when multiple probes were found for a single gene, then expression values of each gene were z-score transformed. Samples with clinical characteristics similar to those in our discovery cohort were then assigned to various groups using the developed predictor. The 70-gene signature^15^, Recurrence Score^16^ and 8-gene Score predictors were calculated for all the samples in the dataset as described previously^14, 17, 72^. Molecular subtype annotation was performed using the Single Sample Predictor described by Hu *et al*.^72, 73^. To apply protein expression-based signatures to gene expression values, per-gene normalization was applied as previously described^17^.

### Statistical analyses and software suites

Survival curves were estimated using a Kaplan-Meier analysis and compared with the log-rank test, using DMFS at 5 years as the end point. Univariate and multivariate Cox proportional hazard analyses were also employed to evaluate the defined prognosis predictors. Correlations were assessed using Pearson’s r and linear regression. The SPSS v16 software package, GraphPad Prism 5.0 and R v2.15.2 (with the *Design* software package 0.2.3) were used for all the statistical analyses. Correlation between node activity and microRNA expression was evaluated using linear regression analyses and Pearson’s correlation. Comparisons between different populations’ characteristics were assessed using Fisher’s exact test, the chi-squared test or the Mann-Whitney test as appropriate. All p-values were two-sided, and p < 0.05 was considered statistically significant. Expression data and network analyses were performed with the MeV and Cytoscape software suites^74, 75^. Class comparison analyses were performed using BRB-ArrayTools v4.2.1.

## Electronic supplementary material

Supplementary information

Supplementary table
