# Supplementary material for: Functional proteomics outlines the complexity of breast cancer molecular subtypes
Source: Sci Rep. 2017 Aug 30;7:10100. doi: 10.1038/s41598-017-10493-w (PMC5577137; doi:10.1038/s41598-017-10493-w)
Supplement: Supplementary file 1 — Supplementary information [file 41598_2017_10493_MOESM1_ESM.pdf]

Supplementary Information

Functional proteomics outlines the complexity of breast cancer molecular subtypes

Angelo Gámez-Pozo[1], Lucía Trilla-Fuertes[2], Julia Berges-Soria[1], Nathalie Selevsek[3], Rocío López-Vacas[1], Mariana Díaz-Almirón[4], Paolo Nanni[3], Jorge M. Arevalillo[5], Hilario Navarro[5], Jonas Grossmann[3], Francisco Gayá Moreno[4], Rubén Gómez Rioja[6], Guillermo Prado-Vázquez[1], Andrea Zapater-Moros[1], Paloma Main[7], Jaime Feliú[8], Purificación Martínez del Prado[9], Pilar Zamora[8], Eva Ciruelos[10], Enrique Espinosa[8], and Juan Ángel Fresno Vara[1*].

[1]Molecular Oncology & Pathology Lab, Instituto de Genética Médica y Molecular-INGEMM, Hospital Universitario La Paz-IdiPAZ, Madrid, Spain. [2]Biomedica Molecular Medicine SL, Madrid, Spain. [3]Functional Genomics Centre Zürich, University of Zürich/ETH Zürich, Zürich, Switzerland. [4]Biostatistics Unit, Hospital Universitario La Paz, Madrid, Spain Department of Statistics, [5]Operational Research and Numerical Analysis, University Nacional Educacion a Distancia (UNED). [6]Medical Laboratory Service, Instituto de Investigación Hospital La Paz-IdiPAZ, Madrid, Spain. [7]Department of Statistics and Operations Research, Faculty of Mathematics, Complutense University of Madrid, Madrid, Spain. [8]Medical Oncology Service, Hospital Universitario La Paz -IdiPAZ, Madrid, Spain. [9]Medical Oncology Service, Basurto Hospital, Bilbao, Spain. [10]Medical Oncology Service, Instituto de Investigación Hospital Doce de Octubre-i+12, Madrid, Spain. [*]Corresponding Author (email address: juanangel.fresno@salud.madrid.org).

Supplementary Figure 1

Chart of samples used in each analysis.

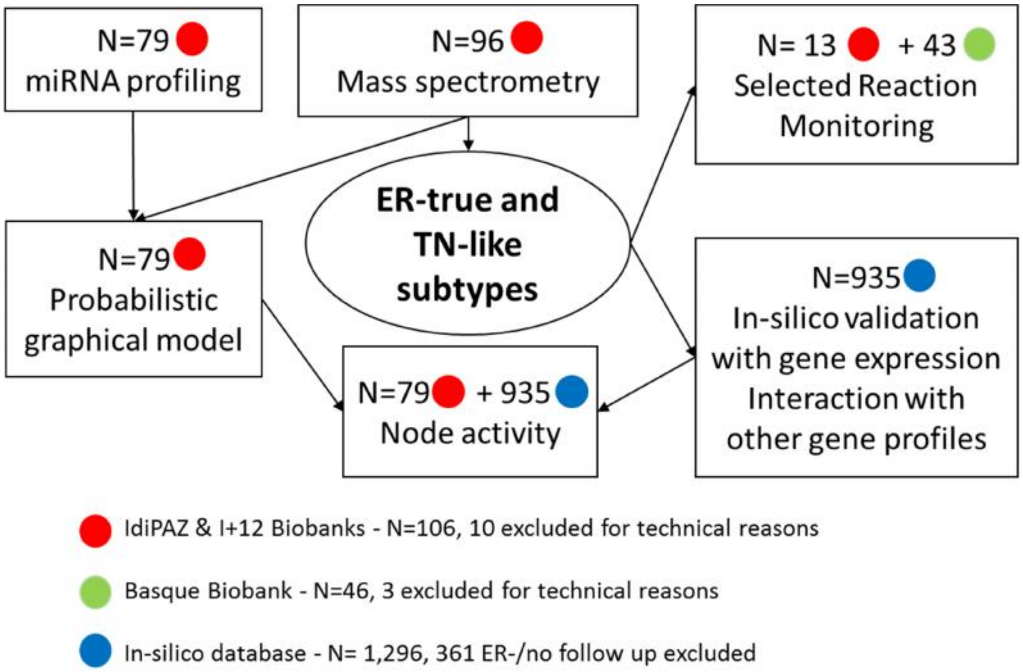

Supplementary Figure 2

Boxplots showing most relevant proteins differentially expressed between ER-true and TN-like tumors. In all cases, n(ER-true)=50, n(TN-like)=21, p< 0.0001.

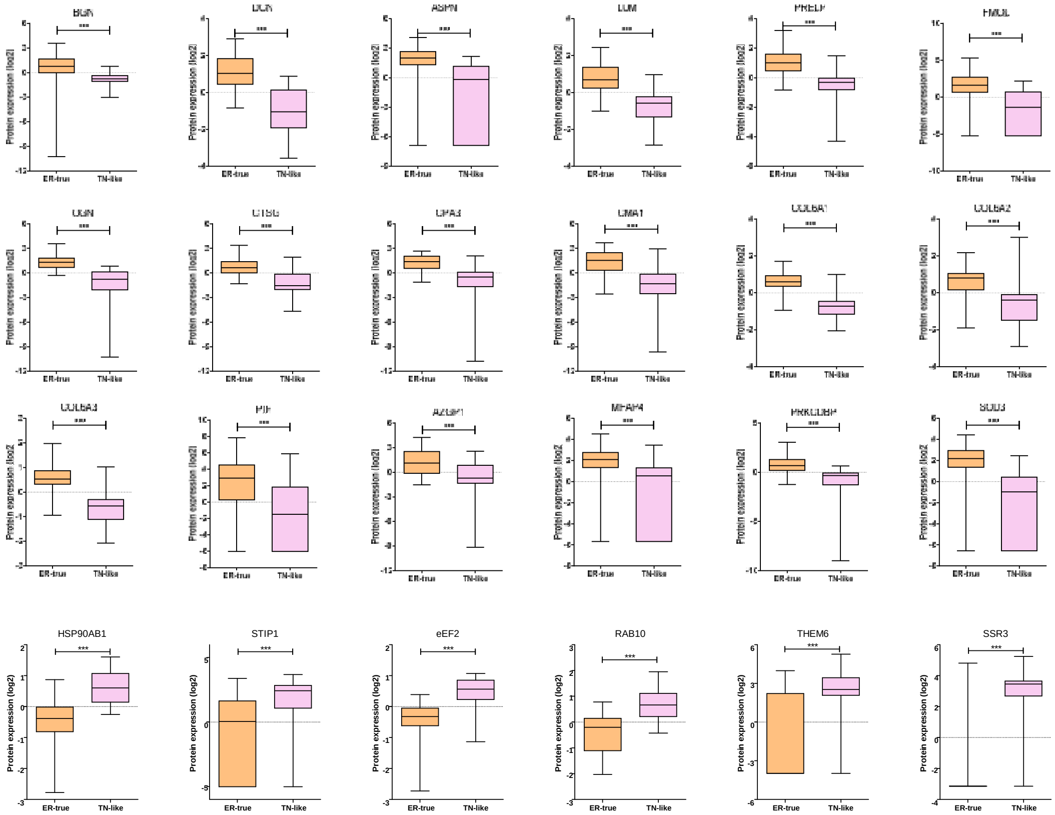

Supplementary figure 3

Boxplots showing miRNAs differentially expressed between ER-true and TN-like tumors. In all cases, n(ER-true)=50, n(TN-like)=21, ***p< 0.0001, **p<0.001.

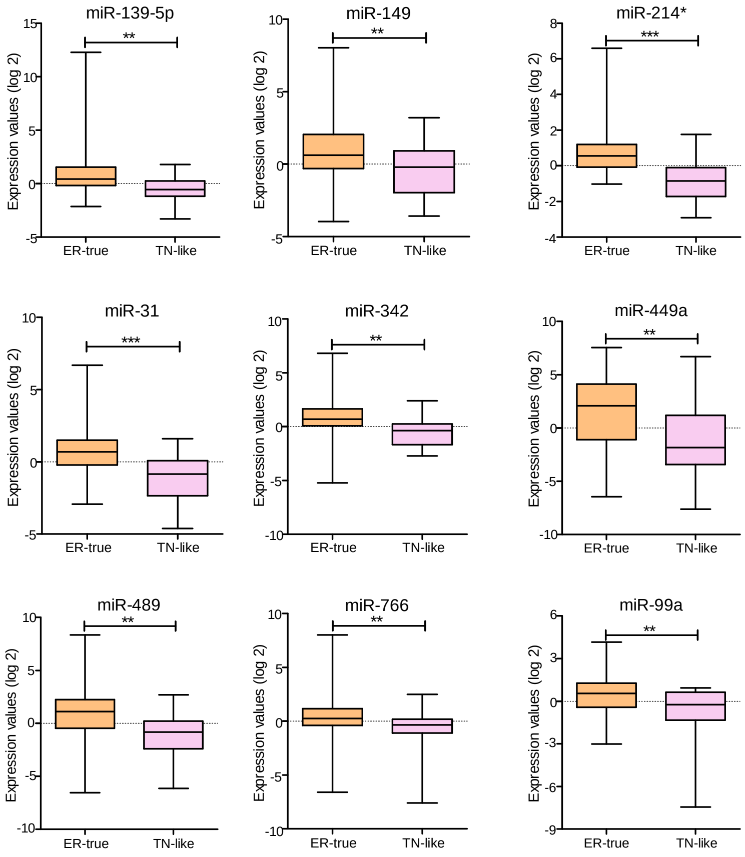

Supplementary figure 4

Boxplots comparing functional node activities between ER-true and TN-like tumors. In all cases, n(ER-true)=50, n(TN-like)=21, ***p< 0.0001, **p<0.001, *p<0.05.

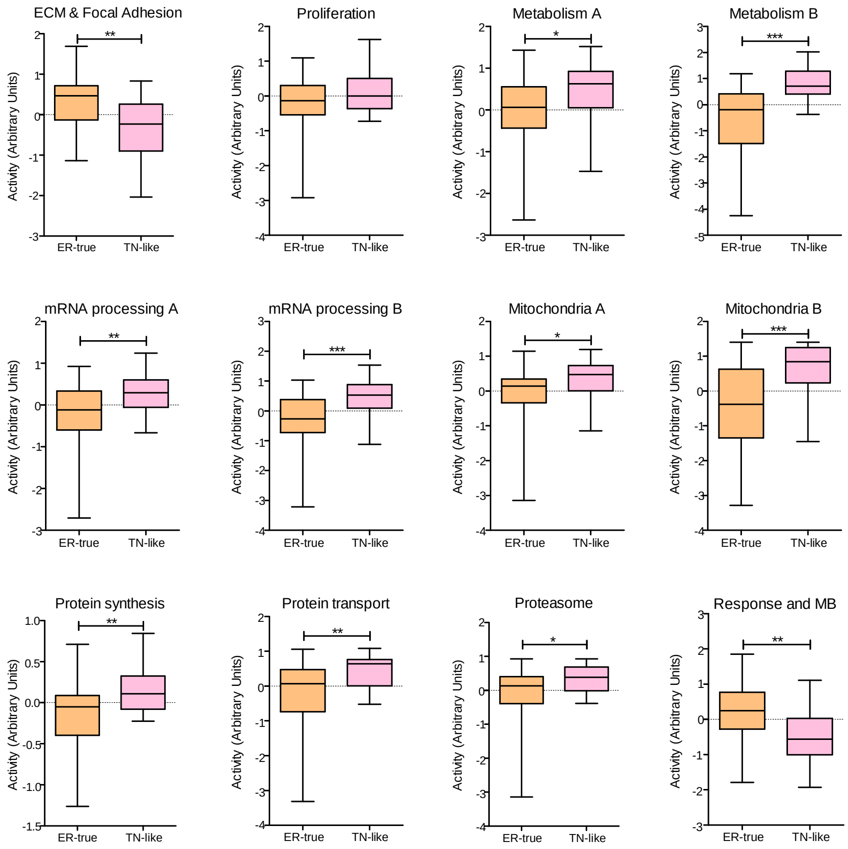

Supplementary figure 5

Correlation between miRNA expression values and functional node activity (n=71).

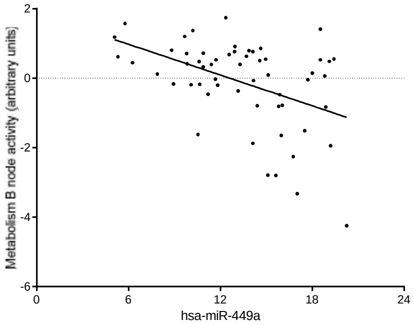

r	-0.4489
95% confidence interval	-0.6352 to -0.2133
R squared	0.2015
P value	
P (two-tailed)	0.0005

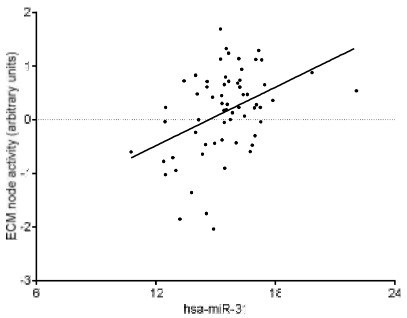

r	0.4124
95% confidence interval	0.1834 to 0.5990
R squared	0.1701
P value	
P (two-tailed)	0.0008

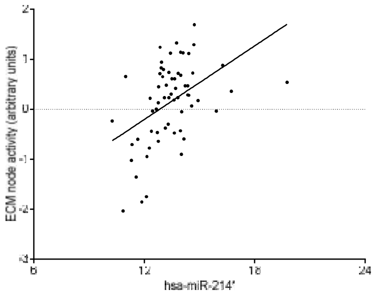

r	0.4538
95% confidence interval	0.2321 to 0.6307
R squared	0.2059
P value	
P (two-tailed)	0.0002

Supplementary figure 6

SRM data hierarchical cluster analysis shows differences between ER-true (ER) and TN-like (TL) tumors.

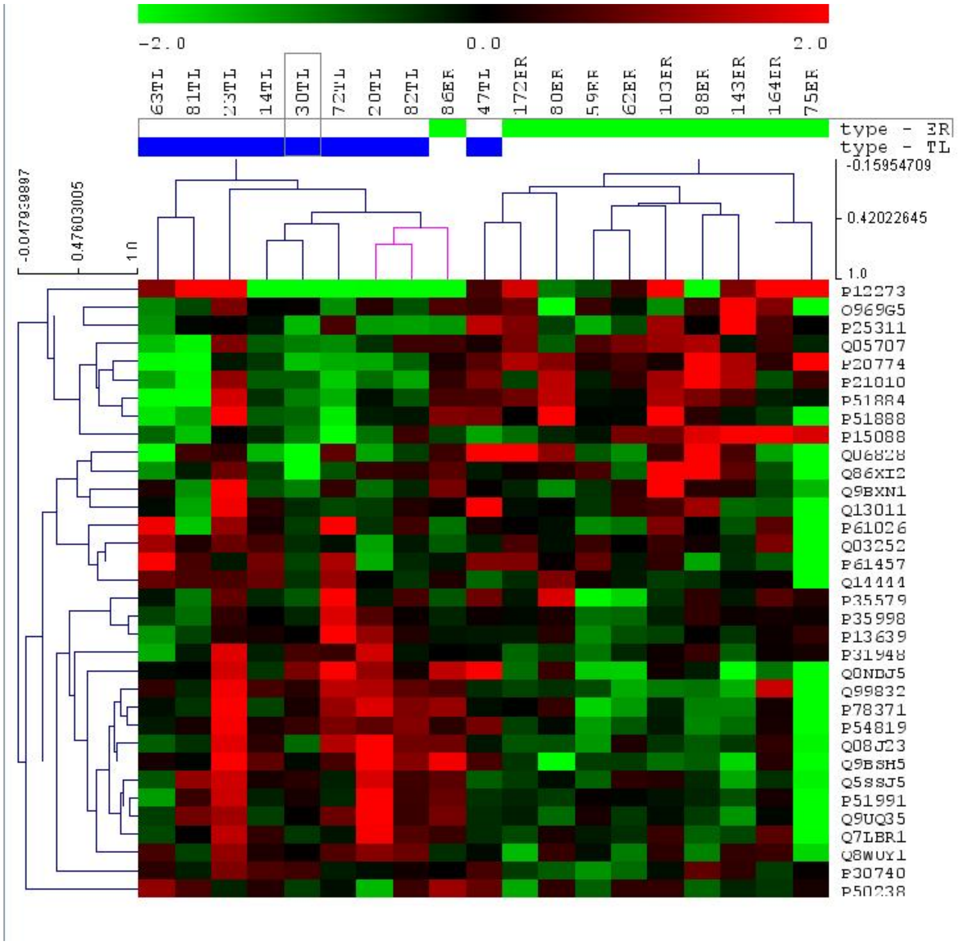

Supplementary figure 7

Kaplan-Meier analysis of 421 tumors with ER+/N+ characteristics from meta-genomics external dataset. DMFS at five years was81.8% and 72.5% for ER-true (n=246) and TN-like (n=164) groups respectively (p<0.005, HR= 0.5769).

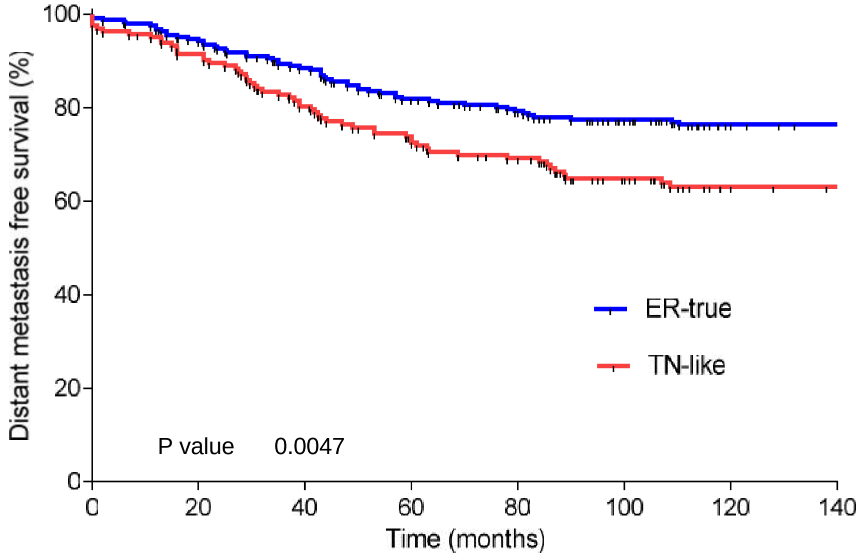

Supplementary Table 1

Proteins differentially expressed between ER+ and TNBC tumors identified by SAM analysis with a FDR<5%.

(see supplementary_table1.xlsx)

Supplementary Table 2

Proteins differentially expressed between ER-true and TN-like tumors identified by SAM analysis with a FDR<5%.

Entry	UNIPROT_ID	Protein name	Gene name	Protein-to-gene
**O14558**	HSPB6_HUMAN	Heat shock protein beta-6	HSPB6	yes
**O15355**	PPM1G_HUMAN	Protein phosphatase 1G	PPM1G	
**P02452**	CO1A1_HUMAN	Collagen alpha-1(I) chain	COL1A1	yes
**P02743**	SAMP_HUMAN	Serum amyloid P-component	APCS PTX2	yes
**P05023**	AT1A1_HUMAN	Sodium/potassium-transporting ATPase subunit alpha-1	ATP1A1	
**P07585**	PGS2_HUMAN	Decorin (Bone proteoglycan II)	DCN	yes
**P08238**	HS90B_HUMAN	Heat shock protein HSP 90-beta	HSP90AB1	yes
**P08294**	SODE_HUMAN	Extracellular superoxide dismutase [Cu-Zn]	SOD3	yes
**P08311**	CATG_HUMAN	Cathepsin G	CTSG	yes
**P12109**	CO6A1_HUMAN	Collagen alpha-1(VI) chain	COL6A1	yes
**P12110**	CO6A2_HUMAN	Collagen alpha-2(VI) chain	COL6A2	yes
**P12111**	CO6A3_HUMAN	Collagen alpha-3(VI) chain	COL6A3	yes
**P12273**	PIP_HUMAN	Prolactin-inducible protein	PIP	yes
**P13639**	EF2_HUMAN	Elongation factor 2 (EF-2)	EEF2	yes
**P15088**	CBPA3_HUMAN	Mast cell carboxypeptidase A	CPA3	yes
**P20774**	MIME_HUMAN	Mimecan	OGN	yes
**P21810**	PGS1_HUMAN	Biglycan	BGN	yes
**P23946**	CMA1_HUMAN	Chymase	CMA1	yes
**P25311**	ZA2G_HUMAN	Zinc-alpha-2-glycoprotein	AZGP1	yes
**P31948**	STIP1_HUMAN	Stress-induced-phosphoprotein 1	STIP1	yes
**P35998**	PRS7_HUMAN	26S protease regulatory subunit 7	PSMC2	yes
**P51884**	LUM_HUMAN	Lumican	LUM	yes
**P51888**	PRELP_HUMAN	Prolargin	PRELP	yes
**P55083**	MFAP4_HUMAN	Microfibril-associated glycoprotein 4	MFAP4	
**P61026**	RAB10_HUMAN	Ras-related protein Rab-10	RAB10	yes
**Q05707**	COEA1_HUMAN	Collagen alpha-1(XIV) chain	COL14A1	yes
**Q06828**	FMOD_HUMAN	Fibromodulin	FMOD	yes
**Q08J23**	NSUN2_HUMAN	tRNA (cytosine(34)-C(5))-methyltransferase	NSUN2	yes
**Q14444**	CAPR1_HUMAN	Caprin-1	CAPRIN1	yes
**Q15459**	SF3A1_HUMAN	Splicing factor 3A subunit 1	SF3A1	yes
**Q4G0X9**	CCD40_HUMAN	Coiled-coil domain-containing protein 40	CCDC40	yes
**Q86XI2**	CNDG2_HUMAN	Condensin-2 complex subunit G2	NCAPG2	yes
**Q8NBJ5**	GT251_HUMAN	Procollagen galactosyltransferase 1	COLGALT1	yes
**Q8WUY1**	THEM6_HUMAN	Mesenchymal stem cell protein DSCD75	THEM6	yes
**Q969G5**	PRDBP_HUMAN	Protein kinase C delta-binding protein	PRKCDBP	yes
**Q99832**	TCPH_HUMAN	T-complex protein 1 subunit eta	CCT7	yes
**Q9BXN1**	ASPN_HUMAN	Asporin	ASPN	yes
**Q9BY44**	EIF2A_HUMAN	Eukaryotic translation initiation factor 2A	EIF2A	yes
**Q9UNL2**	SSRG_HUMAN	Translocon-associated protein subunit gamma	SSR3	
**Q9UQ35**	SRRM2_HUMAN	Serine/arginine repetitive matrix protein	SRRM2	yes

Supplementary Table 3

Gene ontology terms enriched in the protein list differentially expressed between ER-true and TN-like tumors.

Term	Term	p-value
GOTERM_CC_FAT	GO:0031012~extracellular matrix	1.79E-12
GOTERM_CC_FAT	GO:0005581~collagen	2.06E-06
GOTERM_BP_FAT	GO:0030198~extracellular matrix organization	8.84E-05
KEGG_PATHWAY	hsa04512:ECM-receptor interaction	8.59E-04
GOTERM_BP_FAT	GO:0007155~cell adhesion	0.0203
GOTERM_BP_FAT	GO:0022610~biological adhesion	0.0204
KEGG_PATHWAY	hsa04510:Focal adhesion	0.0102
PANTHER_PATHWAY	P00034:Integrin signaling pathway	0.0031

Supplementary table 4

Cox regression analysis of TN-like subtype in each risk category defined by prognostic signatures.

Prognostic test	Risk group	TN-like subtype	N	DMFS	HR	p-value
70-gene Signature	Low	ER-true	449	92.5%	2.1	0.0056
		TN-like	137	84.1%		
	High	ER-true	154	71.0%	1.1	0.58
		TN-like	195	67.1%		
Recurrence Score	Low	ER-true	358	93.7%	2.5	0.0037
		TN-like	114	84.5%		
	Intermediate	ER-true	120	83.7%	0.99	0.97
		TN-like	108	80.1%		
	high	ER-true	125	70.8%	1.22	0.31
		TN-like	143	65.8%		
8-gene Score	Low	ER-true	445	91.5%	2.1	0.0014
		TN-like	165	81.2%		
	High	ER-true	158	74.1	1.3	0.15
		TN-like	167	67.1		
